# Potential Uses of Wild Germplasms of Grain Legumes for Crop Improvement

**DOI:** 10.3390/ijms18020328

**Published:** 2017-02-04

**Authors:** Nacira Muñoz, Ailin Liu, Leo Kan, Man-Wah Li, Hon-Ming Lam

**Affiliations:** 1 Centre for Soybean Research of the Partner State Key Laboratory of Agrobiotechnology and School of Life Sciences, The Chinese University of Hong Kong, Hong Kong, China; munioz.nacira@inta.gob.ar (N.M.); merestarry@gmail.com (A.L.); leokan@link.cuhk.edu.hk (L.K.); limanwah@cuhk.edu.hk (M.-W.L.); 2 Centro de Investigaciones Agropecuarias-INTA, Instituto de Fisiología y Recursos Genéticos Vegetales, Córdoba X5000, Argentina; 3 Cátedra de Fisiología Vegetal, Facultad de Ciencias Exactas Físicas y Naturales, Universidad Nacional de Córdoba, Córdoba X5000, Argentina

**Keywords:** domestication, genetic bottleneck, genetic diversity, genomics-assisted breeding, grain legumes, wild germplasms

## Abstract

Challenged by population increase, climatic change, and soil deterioration, crop improvement is always a priority in securing food supplies. Although the production of grain legumes is in general lower than that of cereals, the nutritional value of grain legumes make them important components of food security. Nevertheless, limited by severe genetic bottlenecks during domestication and human selection, grain legumes, like other crops, have suffered from a loss of genetic diversity which is essential for providing genetic materials for crop improvement programs. Illustrated by whole-genome-sequencing, wild relatives of crops adapted to various environments were shown to maintain high genetic diversity. In this review, we focused on nine important grain legumes (soybean, peanut, pea, chickpea, common bean, lentil, cowpea, lupin, and pigeonpea) to discuss the potential uses of their wild relatives as genetic resources for crop breeding and improvement, and summarized the various genetic/genomic approaches adopted for these purposes.

## 1. Introduction

Wild plants have been domesticated for thousands of years since the beginning of human civilization, as a means to ensure a stable food supply. Through plant breeding activities over the centuries, crop plants have been manipulated to develop new and desirable traits [1]. The artificial selection processes based on phenotypes (Appendix A) drove the development of new varieties with desirable features and are considered the most ancient form of plant breeding. Over time, these new species or varieties have become genetically diverged from their original progenitors.

Challenged by the demand of the ever-increasing global population [2], the negative effects of mono-cropping and climate change [3], there is constant need for crop improvement. Furthermore, the genetic diversity of crops is generally low due to a strong bottleneck effect during domestication and artificial selection, hence limiting the potential for crop improvement [4].

Wild relatives are potential genetic resources for crop improvement [5,6,7], as well as for exploring new or alternative production systems. The rationale is straightforward: wild populations must contain higher genetic variability as they were propagated in a wide range of habitats without human selection [8]. Just to demonstrate this point, desirable traits such as biotic and abiotic stress resistances and special nutritional values important for crop improvement can be found in some of the wild relatives [9,10]. On the other hand, since the genetic modification of food crops is still controversial among the public, it is more acceptable to introduce genetic materials from wild relatives (of the same or closely related species) into crop varieties through breeding, hybridization or some other techniques [11] to generate improved crops. Although the use of wild relatives as sources of new alleles (Appendix A) is challenging due mainly to the linkage drag (Appendix A), advances in genetic and genomic researches of crop plants and their wild relatives have expanded our understanding on complex traits and led to the discovery of new genes.

The idea of generating exotic genetic libraries in order to accelerate plant breeding is further supported by the development of genomic and genetic tools in the post genomic era [12]. Nonetheless, many genetic populations generated over the years have not been further genotyped or phenotyped under different environmental conditions. It is also important to point out that wild genetic materials are not always available, and therefore establishing formal holding institutions for each crop worldwide, together with constant selection and conservation of this biodiversity, should be a priority. Two recent review papers have summarized the main repository institutions in the world for grain legumes as well as the number of introductions and collections of both cultivated and wild pulses [13,14].

Molecular plant breeding has been hailed as the foundation of 21st-century crop improvement and it integrates traditional plant breeding practices, molecular markers, genomic research and biotechnology [15]. Undoubtedly, the development of tools and strategies over the past 30 years has contributed multi-dimensionally to crop improvement and the information generated is huge and complex. However, one should note that the success of any breeding program for crop improvement depends on the plant material, and specifically on its variability for key traits.

Grain legumes refer to legumes the seeds of which are harvested for consumption. Commonly known grain legumes include soybean, peanut, pea, chickpea, common bean, lentil, cowpea, lupin and pigeonpea. The current agronomic challenge is the generally lower yield in grain legumes compared to cereals, together with the difference between their role as a diet staple for certain populations and the geographical locations where they are grown [13]. However, unlike cereals, most of the grain legumes are good sources of protein which make them good substitutes for animal proteins. The seeds of grain legumes are also sources of edible oils and other compounds of high nutraceutical values [16]. Another unique feature of legumes is that they are able to interact with soil rhizobium to fix atmospheric nitrogen. Such a mutualistic relationship provides nitrogen to support the growth of the legume plant and helps replenish soil fertility, thus minimizing the need for inorganic nitrogen fertilizers. As a result, legume is usually used in crop rotation or intercropping practices to improve diversity, quality and sustainability in traditional food production systems, providing an excellent solution for increasing agro-ecosystem services.

In this review, we will focus our discussions on grain legumes as 2016 has been declared the International Year of Pulses by the United Nations. We will include a brief history of their domestication, a description on the impacts of genome sequencing on the studies of wild relatives through the generation of genetic maps, the use of the new strategy of genotyping-by-sequencing (GBS) and the search and introgression of wild alleles in breeding programs. Finally, we will discuss our perspectives on the roles of wild relatives in the new challenges that arise in agriculture.

## 2. A Brief History of the Domestication of Major Grain Legumes

Grain legumes were planted as companion crops of wheat and barley when agriculture began in the Near East [17,18], while some other important grain legumes have their origins of domestication in Asia and the New World. The domestication of plants was generally associated with centers of cultural diversity along with fascinating relationships between ancient human settlements and particular phytogeographic characteristics. It is difficult to trace back the history of domestication, but today a significant amount of information generated by agronomists, biologists, anthropologists and historians has been made available to support the community hypothesis on the centers of origin and domestication. These descriptions painted a complicated picture of the domestication process through the associations with different ancient civilizations, such as how it was done before and after the existence of seed exchanges for cultivation, natural or intentional crossbreeding with wild relatives in different parts of the world, etc. In this section, we will summarize the existing information about the centers of origin and domestication of grain legumes analyzed in this review. This information allows us to grasp their importance in ancient times, understand their history of domestication and identify potential geographical locations of the divergence from their wild ancestors for future improvement. In addition, the concept of “gene pool” proposed by Harlan and de Wet (1971) [19] is particularly important for using wild relatives for crop improvement. The classification of crop related species is not based on formal taxonomy, but on gene pools with different levels (possibilities) of crosses. Primary, secondary and tertiary “gene pools” refer to subspecies or species that can, respectively: (i) freely cross with crops to produce fertile hybrids; (ii) cross with crops to produce a degree of fertile hybrids; and (iii) cross with crops only by using special approaches such as chromosome doubling, embryos rescue, tissue culture, etc.

Soybean (*Glycine max* L.): Soybean is one of the oldest crops [20]. The cultivated soybean was domesticated from an endemic wild species in China, *Glycine soja* Siebold & Zucc., probably 6000–9000 years ago [21]. Theodore Hymowitz, an eminent researcher in the history of soybean, has suggested that it is unlikely we will ever know the exact time when soybean cultivation began, and, based on early bronze inscriptions, domestication may have occurred during the Shang Dynasty (1500–1100 B.C.) [22]. There is evidence that soybean appeared to be domesticated during Zhou dynasty in northeastern China [22], corresponding to Vavilov’s Chinese–Japanese center [20]. The earliest documented evidence of *Glycine* spp. use by humans came from a Neolithic site 7800–9000 years ago in Jiahu, Henan Province, where charred remains of soybean were recovered [21].

Peanut (*Arachis hypogaea* L.): The center of origin of *Arachis* spp. is South America, with wild species found in Bolivia, Brazil, Paraguay, Argentina and Uruguay. The oldest archaeological records of *A. hypogaea* came from Huarmey Valley, Peru, dating from approximately 3500–4500 years ago [23,24], although there is evidence suggesting that peanut could have originated in northern Argentina and eastern Bolivia [25]. There are also records of peanut use by ancient people in Ñanchoc Valley in northern Peru approximately 7840 years ago, although these records may correspond to the use of wild species or the very early stage of domestication [26]. Archaeological together with genetic evidence suggests that *A. montícola* Krapov. & Rigoni is the tetraploid wild ancestor from which the peanut was domesticated [26,27], in a complex scenario which involved natural evolution and human domestication of diploid species distributed in Argentina and Bolivia [27]. Important evidence suggests that two diploid wild species, *Arachis duranensis* Krapov. & W.C.Greg. (AA) and *Arachis ipaensis* Krapov. & W.C.Greg. (BB), are the progenitors of cultivated peanut. A single hybridization event between the two progenitors followed by genome duplication about 3500 years ago led to the origin of cultivated peanut [28,29].

Pea (*Pisum sativum*): *Pisum sativum* L. has its origin and domestication in the Mediterranean, primarily in the Middle East, about 10,000 years ago [30,31]. There are currently thousands of pea varieties from hundreds of years of selection and breeding. The natural growing range of the wild representatives of *P. sativum* (*Pisum elatius* M. Bieb. and *Pisum humile* Boiss. & Noë) extends from Iran and Turkmenistan through Anterior Asia, northern Africa and southern Europe. However, it is difficult to precisely locate the diversity center given the early domestication and the diverse areas of cultivation [32]. Three pea types are currently recognized: (i) *Pisum sativum*, which extends from Iran and Turkmenistan through Anterior Asia, northern Africa and southern Europe; (ii) *P. fulvum* Sibth. & Sm., which is found in Jordan, Syria, Lebanon and Israel; and (iii) *P. abyssinicum* A. Braun, which is found from Yemen to Ethiopia [33]. It was suggested that both *P. sativum* and *P. fulvum* were domesticated in the Near East about 11,000 years ago from an extinct ancestor of *Pisum* spp., and *P. abyssinicum* was developed from *P. sativum* independently in Old Kingdom or Middle Kingdom Egypt about 4000–5000 years ago [34].

Chickpea (*Cicer arietinum* L.): Chickpea was domesticated from the wild progenitor, *C. reticulatum* Ladiz., known from southeastern Turkey and adjacent Syria about 11,000 years ago [35,36]. It is interesting that, unlike other legumes, the center of origin of the wild progenitor is confined to a small specific area. Domesticated chickpeas have been found in archaeological sites corresponding to the Pre-Pottery Neolithic period and is one of the “founder crops package” that gave rise to farming [30]. Two main chickpea varieties are currently cultivated worldwide: the small-seeded *desi* and the larger-seeded *kabuli* [37].

Common bean (*Phaseolus vulgaris* L.): Most *Phaseolus* species of small seed and leaf were originated from Mesoamerican [38,39,40]. Common bean was domesticated in Mesoamerica and in the Andes about 5000 years ago and an important number of investigations have revealed and followed the complex evolutionary and domestication history of the different genetic pools [41,42,43]. Interestingly, there is a wide variety of common bean, all belonging to the same species and are classified as “landraces” with a fascinating diversity of seed sizes, shapes and colors. They are the results of a complex and magnificent history of domestication and selection. While *P. vulgaris* is the most economically important species of *Phaseolus*, there are other species of the genus that have been domesticated: *P. lunatus* L., *P. dumosus* Macfad., *P. coccineus* L. and *P. acutifolius* A. Gray.

Lentil (*Lens culinaris* Medik.): Lentil is one of the oldest crops cultivated and domesticated by humans on the planet and has been recovered from archeological sites dating from the Neolithic period [44]. Lentils were domesticated in the Near East in an area called “the cradle of agriculture” about 11,000 B.C. [45] from the wild progenitor, *Lens culinaris* subsp. *orientalis* (Boiss.) Ponert.

Cowpea (*Vigna unguiculata* [L.] Walp.): Cowpea is widely cultivated in the semiarid and sub-humid zones of Africa and Asia as one of the most important food for sub-Saharan populations, adaptable to marginal and changing environments [14]. With sparse evidence, the history of cowpea domestication remains to be elucidated, complicated by the diverse morphology and widespread distribution of the wild species [46]. Central-southern Africa seems to be the center of origin of cowpea, and west Africa and India are the first and second most probable centers of domestication respectively [47,48]. The earliest archaeological evidence of cowpea cultivation in Africa dated from 1830–1595 B.C. [46]. The wild progenitor of cowpea is *V. unguiculata* var. *spontanea* (formerly var. *dekindtiana*).

Lupin or lupini bean (*Lupinus* L.): The Mediterranean region and the American continent are two centers of wild lupin and of domestication. It was probably introduced into cultivation in the Old World in ancient Greece. The earliest archaeological evidence of lupin dated from 2000 B.C. in the tombs of Egyptian Pharaohs where domesticated seeds were discovered. Andean pearl lupin (*L. mutabilis*) was domesticated in 6th–7th Century B.C. in America, by a pre-Incan culture in what is modern-day Peru. *L. albus* L. (white lupin), *L. luteus* L. (yellow lupin), *L. angustifolius* L. (narrow-leafed lupin), *L. mutabilis* Sweet (pearl lupin) and *L. polyphyllus* Lindl. (multifoliate or Washington lupin) are currently under widespread cultivation for many different proposes [49].

Pigeonpea (*Cajanus cajan* [L.] Millsp): Populations of the wild progenitor, *Cajanus cajanifolius* (Haines) Maesen, have been identified in eastern Peninsular India alongside a diverse group of other *Cajanus* species. *C. cajanifolius* is rare today, probably due to habitat loss [50,51]. Archaeological evidence suggests that pigeonpea could have been domesticated during the middle of the 2nd Millennium B.C. by settlements in Orissa, close to the areas where the wild species grew (*Cajanus cajanifolius* Gopalpur and Golbai) [51]. Currently, pigeonpea is widely cultivated in all tropical and semitropical regions.

## 3. Plant Domestication: More Than a Syndrome

Loss in genetic diversity due to the founder effect and domestication syndrome are two main characteristics of cultivated crops. Domestication syndrome is defined as all the morpho-physiological modifications that make the cultivated crops different from their wild ancestors, conferring adaptability for agriculture [43,52]. Some of these include changes in growth habits, seed dispersal mechanisms, loss of germination inhibition, etc. These changes seem to have occurred in parallel in different regions of our planet [53]. A conceptual framework has been proposed to distinguish between crucial domestication and crop evolution/diversification traits, i.e., between short episodes and long-term historical processes such as domestication [54]. In this context, it is worth noting that archaeobotany has also characterized and contrasted different patterns of domestication, such as that between legumes and non-legume crops. Regarding the seed size, grain legumes do not show evidence of seed size increase with domestication, although selection pressure persisted for larger seeds in association with animal-drawn ploughs (or ards) [55]. Others have proposed that the seeding depth by humans might have contributed in some circumstances to increasing the biomass of the seed, but this did not seem to have strong empirical support after testing [56]. This shows how agriculture is an interaction between cultural behavior and management practices acting together on the available genetic diversity of plants.

Likewise, the scientific discussion about conscious or unconscious selections is a topic of great importance in relation to plant domestication. If cultivation practices and regimes were strong selection pressures during the domestication of crops, we should also study the desires and decisions of human beings [57]. There is increasing evidence suggesting that humans have actively modified particular ecosystems to increase the availability of certain plant resources hundreds of years before the manifestation of the indicators of domestication [58].

No matter what the situations were, it is encouraging to consider the progress that has been made and what can be foreseen regarding the understanding of the spatio-temporal patterns of domestication, the speed at which it happened, intentionality versus serendipity, etc. [59]. Finally, it is important to keep in mind that the plant domestication process is still occurring at present [60]; that it is not only a series of events from the past. There is still great potential yet, with the unprecedented development of selection tools that would allow us to produce more and higher quality food for our planet.

## 4. Revisiting the Genetic Diversities and Potentials of Wild Relatives of Crop Plants

The release of the reference genome of the dicot model plant *Arabidopsis thaliana* in 2000 [61] and the two rice genomes [62,63] marked the beginning of the age of plant genome sequencing. Nevertheless, the cost of sequencing a crop genome was barely affordable at that time. The advances in next-generation sequencing technologies have largely reduced the sequencing cost and labor required. It is expected that the sequencing cost can be reduced from several million to several thousand US dollars soon [64]. This makes sequencing of crop genomes more accessible to researchers and hence more and more crop genomes are being sequenced in the hope of speeding up crop research. In recent years, in addition to sequencing crop genomes, efforts have also been made to sequence the genomes of their wild relatives (Table 1). The data would facilitate greater understanding of the evolution or domestication relationship between the crops and their wild relatives, while at the same time they would also provide a solid ground for the mining of important genetic resources from the wild species.

Comparative population genomic analyses have confirmed that wild species tend to have higher genetic diversities, making the wild relatives promising natural reservoirs of potential genes/alleles for crop improvement. Wild soybeans have been shown to have higher genetic diversities over cultivated soybeans [78]. In addition to the overall genetic diversity, researchers have also uncovered specific gene sequences unique to wild soybeans that confer enhanced disease resistance and metabolic functions [78], which serve as good candidates for soybean improvement. In contrast, a study using wild soybeans in Korea also identified possible gene loss events in wild species [79]. The discrepancy in these two studies may imply that the outcome of any comparative genomic study on wild germplasms really relies on the diversity of the wild collections. Qi et al. (2014) [66] conducted the de novo sequencing of a wild soybean, *Glycine soja* (*G. soja*) W05 helping to build a deeper understanding of the wild soybean genome and demonstrating the potential of its use for crop improvement. Li et al. (2014) [67] also published the de novo assembly of 7 wild and cultivated soybeans and provide a pan-genome analysis identifying lineage-specific genes, copy number variations and mutations that are eventually associated with positive human selection for certain agronomic traits, making this an important source of information regarding wild soybean genetic diversity. Using a complete re-sequencing approach, Zhou et al. (2015) [80] analyzed the genomic diversities of 302 lines of wild soybeans, landraces and improved varieties. They also characterized important genomic regions associated with domestication and improvement for important agronomic traits using genome wide association studies and discovered that some traits are closely associated with specific geographic regions.

In 2014, a high-quality reference genome of Andean (Peruvian) common bean (*P. vulgaris*) landrace (G19833) was published along with the pooled re-sequencing analysis of 30 wild individuals from the Mesoamerican and Andean populations. The results suggested that the wild Mesoamerican populations are more genetically diverse than those from the Andes. Both populations are also substantially different based on *F_st_* values (fixation index) (Appendix A), and the divergence probably occurred ~165,000 years ago. An interesting contradiction occurred when comparing landraces and wild relatives within each of the two genetic pools. Landraces from Mesoamerica are less diverse than their wild counterparts while the Andean landrace populations are more diverse than their wild Andean relatives [41]. Likewise, the recently published genome assembly of common bean BAT93 with a Mesoamerican origin, together with transcriptomic and phylogenetic analyses, suggests that most of the bean-specific gene family expansions predated the differentiation between Mesoamerican and Andean gene pools and consequently prior to domestication. The latter results suggest that pre-existing adaptations could contribute to the subsequent domestication process [72]. However, a complete analysis of 577 accessions of common bean revealed the existence of several genetic groups and the presence of varying degrees of diversity in Mesoamerica and the Andes based on the genetic-spatial patterns of wild common bean. An interesting landscape genetics approach demonstrated that demographic processes and natural selection are correlated with the characterized genetic structure. This can be a source of potentially important genes associated with the adaptation to specific local environmental conditions [81].

The draft genome sequence of chickpea (*Cicer arietinum* CDC Frontier, a *kabuli* variety) published in 2013 together with the re-sequencing of 29 elite (17 *desi* and 12 *kabuli*) varieties allowed us to get a first glimpse at the genetic history of chickpea accessions [69]. The results suggested that the genetic diversity in the *desi* group was slightly higher than in the *kabuli* group, but population structure, diversity and phylogenetic analyses showed a mixing of *desi* and *kabuli* genotypes (Appendix A) during breeding processes [69]. In the same year, the draft genome sequence of chickpea (*Cicer arietinum* ICC4958, a *desi* variety) was also published [68] and the final version of this genome was released in 2015 [70]. The evidence suggests that the *kabuli*-type chickpea was recently derived from the *desi*-type through artificial selection for increased seed size, from a small gene pool [82]. This hypothesis is supported by results showing that the divergence of the two chickpea types occurred about 8000 years ago. However, an older initial divergence can also be detected about 160,000 to 250,000 years ago [70]. Recently, the SNP (Appendix A)-genotyping of 93 wild and cultivated *Cicer* spp. accessions on a genome-wide scale revealed the natural allelic diversity, population genetic structure, phylogeny, etc., within a wider genetic pool [83]. High intra- and inter-specific polymorphic potential (66%–85%) and broader natural allelic diversity (6%–64%) were described, suggesting a great potential for the discovery of new alleles of importance for specific geographical origin and phenotypic characteristics in the wild *Cicer* primary gene pool [83].

Peanut (*Arachis hypogaea*) is an allo-tetraploid (AABB-type genome; 2*n* = 4*x* = 40) apparently derived from a hybridization between two diploid species and further polyploidization [29,84]. A recent sequencing effort reported the genome sequences of its diploid ancestors, *Arachis duranensis* and *Arachis ipaensis*, carrying the A and B subgenomes. The information generated, together with empirical evidence by the crossing of these two species support the proposed hypothesis of the origin of the cultivated peanut [28]. Armed with this genomic information on the diploid ancestors, the researchers were able to characterize some genomic regions and genes probably associated with disease resistance, and describe important genomic information such as gene evolution, DNA methylation, and transposons, thus laying the foundation for more in-depth peanut genomics studies.

Since the initiation of the pigeonpea genome project [85], a concerted international effort has been made to elucidate the first draft genome of pigeonpea. The cultivated pigeonpea genome information shows extensive synteny between pigeonpea and other legumes, including those belonging to different clades but lacking the recent genome duplication as that found in soybean [76]. Population structure analyses using SNPs from 79 pigeonpea accessions and 31 wild relatives disclosed information about its domestication history and relationships with wild species. Evidence suggests that the recent gene flow between cultivated and non-cultivated forms occurred probably as a result of frequent cross-pollination between this diploid crop and its wild relatives. The gene pool of wild pigeonpea shows high genetic diversity and the presence of rare alleles is potentially important for crop improvement [86].

Sequencing the genomes of lentil, pea and cowpea are being undertaken by the scientific community. Lentil Genome v1.2 is available in a pre-release form [87]. Pea genome data are now available at the Unité de Recherche Génomique Info (URGI; [88]). The elucidation of the cowpea genome is moving forward by the Cowpea Genomics Initiative (CGI; [89]) and important progress has been achieved to understand the genetic diversity among *Vigna* species, mainly by using traditional markers [90,91,92,93,94]. A recent work using genotyping-by-sequencing of globally cultivated cowpea genotypes (768 genotypes from 56 countries) revealed the worldwide distribution of genetic diversity and structures, suggesting the existence of three genetically well-differentiated populations associated with areas where the genotypes were collected, supporting the hypothesis of two areas of domestication: West and East Africa as the first and India as a sub-domestication region of cowpea [95]. A previous report also showed extensive gene flows between wild and domesticated types [96].

Lupin is an interesting complex of species of the genus *Lupinus*, and its domestication and cultivation have origins in both the New and the Old World. There are more wild germplasms that the cultivated ones, and the main repository institutions are summarized in an excellent review [14]. However, much work still needs to be done to research the genomic information for all lupin species. The lupin genomes vary widely in terms of chromosome numbers. The taxonomy has been confusing but it is being improved constantly [14,49,97,98,99]. Different morphological and ecological adaptations are associated with lupins from the New World versus those from the Old World [100]. Given the current importance of *L. angustifolius*, a draft genome of the narrow-leafed cultivar Tanjil has recently been published, providing useful information for understanding the genetic basis in the genistoid clade of *Papilionoideae* legumes and for facilitating genomics-based breeding approaches [74].

Complete genome releases and constant genome quality improvement will undoubtedly accelerate the improvement of important traits of cultivated grain legumes [101,102]. The availability of genomic information on incompletely sequenced grain legumes is critical, considering that many of them are important staples in many poor countries. Therefore more time and money should be invested in increasing our knowledge of these neglected grain legumes, in order to facilitate the improvement of specific features suitable for very small family farmers and/or alternative production systems [13].

The genome information of both the crops and their wild relatives have also served as the foundation for studies such as comparative genomics, functional genomics (transcriptomics, proteomics and epigenomics), association mapping (Appendix A) and gene discovery. High-density markers generated by sequencing are important for precise marker-assisted selections (Appendix A) [103] of targeted beneficial genomic regions and the removal of undesirable regions carried over from the wild species. Furthermore, genome-wide high-density markers are also important for genomic selections [104] and the current unprecedented availability of technologies and genomic information should have a significantly positive impact on the quality, diversity and speed of breeding programs. The major breeding strategies of grain legumes, taking into consideration the genome size, ploidy, genome availability and number of accessions in the main holding institutes in the world, have been recently summarized [13,14].

In the following section, we will discuss the population approaches for dissecting the genetic variabilities and looking for important genomic regions associated with traits for nine grain legumes, especially focusing on the use of wild relatives as potential reservoirs of variability.

## 5. Traditional and Sequencing-Based Genetic Mapping Using Wild Relatives

In the past, through genetic mapping, we could locate the approximate positions of target loci represented by the distance from gene markers in cM. To identify the target gene, a large genetic population is needed to pinpoint a small region in the genome. BAC (bacterial artificial chromosome) sequencing or primer walking may be needed to identify the genes linked to the genetic markers. With genome sequencing, the situation has improved. First, the physical positions of markers can be found in well annotated reference genome. Secondly, sequences and gene models within the locus of interest can be examined to pinpoint possible gene candidates for more in-depth studies. Thirdly, by examining the genomic sequences, non-synonymous SNPs, InDels, and CNVs can be discovered, which can then be used to explain the phenotypic differences.

**Figure 1:**
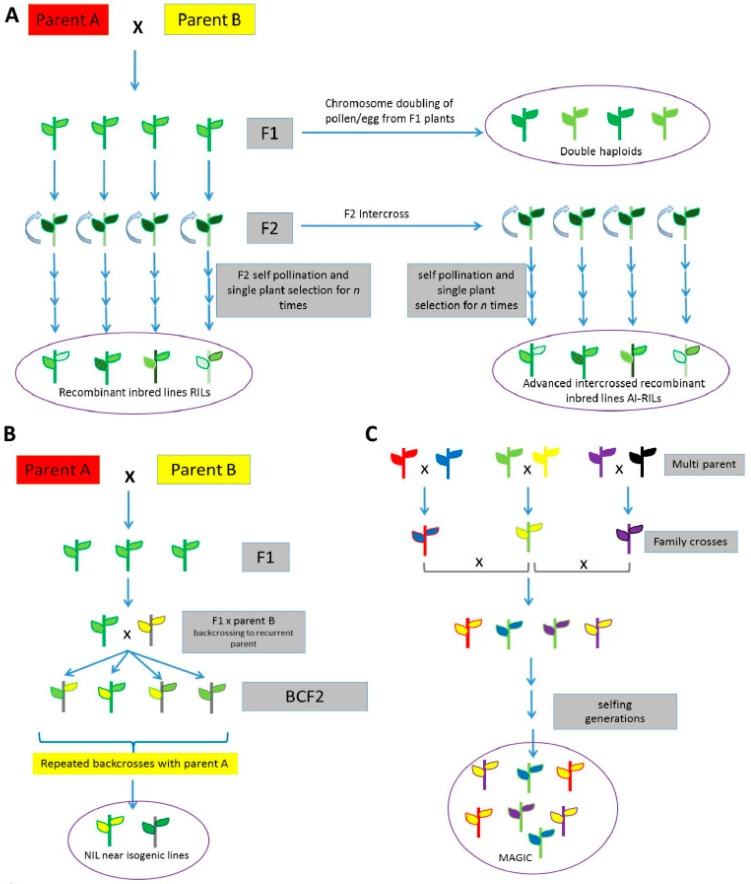
Population approaches commonly used in genetic studies. Marker-assisted backcrossing or gene pyramiding is the most successful method using genomics information, and are specially used in the introgression (Appendix A) of wild traits. For the identification of genomic regions/genes associated with a certain trait, three major mapping strategies are being used: QTL mapping (linkage mapping), association mapping (linkage disequilibrium mapping) and joint linkage-association mapping (Appendix A). Here are the main population approaches based on initial: bi-parental (**A**,**B**); or multi-parental (**C**) crosses for mapping. (**A**) F_2_: Individual F_1_ plants are self-pollinated to produce an F_2_ population; Doubled haploids (DH): genotypes formed when haploid cells (pollen/egg) are subjected to chromosome doubling. Recombinant inbred lines (RILs): a population generated from F_2_ individual plants that are repeatedly self-pollinated *n* times. Advanced intercrossed recombinant inbred lines (AI-RILs): a population generated by randomly and sequentially intercrossing the F_2_ lines followed by repeated self-pollinations *n* times; (**B**) Backcross inbred lines (BILs): repeated backcrossing (Appendix A) of F_1_ lines with one of its parents; Near isogenic lines (NILs): backcrossing of F_1_ with recurrent parent using lines that are identical except for differences in a few genetic loci; and (**C**) Multi-parent advanced generation inter-cross (MAGIC): a population produced by intercrossing families from multiple parents, followed by self-pollination *n* times.

An advantage of using wild crop relatives over the use of unrelated species is that the former is more likely to produce fertile offspring with the domesticated crops for generating mapping populations or for breeding purposes. Genetic mapping can involve the generation of different kinds of populations, such as unrelated populations (mini-core collections), advanced backcross populations (A-BC) [105], recombinant inbred lines (RILs) [106], near isogenic lines (NILs) [107], nested association mapping (NAM) populations [108] and multi-parent advanced generation inter-cross (MAGIC) population [109], and so on. Each method has its own advantages and serves a unique purpose (Figure 1) [110].

Wild relatives have played unique roles in association mapping. In general, crop genomes have long linkage disequilibrium (LD) half-lives [78,111,112,113]. The resolution of genome-wide association mapping (GWAS) is usually dependent on the size of the LD block. Therefore, the resolution of maps generated from cultivated varieties alone tends to be low owing to the low LD decay rate. Due to the higher genetic diversity and probably higher outcrossing rate among wild germplasms, the genomes of the wild relatives usually have higher linkage disequilibrium decay rates [78,111,112,113], and thus they can serve as better materials for GWAS compared to the cultivated crops.

On the other hand, it has also been demonstrated that some QTLs (Appendix A) fixed by domestication can hardly be mapped using cultivated populations. For example, two 100-seed weight loci on Chromosome 12 of the soybean genome suggested to be related to domestication were only found in the wild soybean-derived populations and not the cultivated soybean-derived populations [114]. Hence, mapping involving wild relatives may help discover more domestication-related loci which may also be important for crop improvement.

Genetic mapping is important for the identification of genes/loci controlling specific agronomic traits. There have been a lot of mapping studies using wild crop relative-derived populations. In this review, we specifically collected all the information about population approaches in pulses. For any crop, the variability in the genomes of the wild relatives is high and thus provides a higher number of genetic markers for mapping. In addition to the traditional markers such as variable length polymorphisms, variable number of repeats, InDel markers and SNPs, there is currently an increased reliance on array-based and sequencing-based markers such as Diversity Arrays Technology (DART) [115], restriction site-associated DNA markers (RAD) [116], reduced-representation libraries (RRLs), complexity reduction of polymorphic sequences (CRoPS) [117], bin markers [118] and other new technologies [119]. Meanwhile, whole-genome sequencing has played a pivotal role in genetic mapping. In theory, whole-genome sequencing can generate the highest density of markers. While array-based SNP detection is limited by the number of probes on the array, as long as an SNP is covered by sequencing reads, it can be used as a marker. However, in reality, constrained by the sequencing depth (a factor of the operation cost) and the high error rates of next-generation sequencing, a “bin”, which is an array of high-confidence SNPs detected by a sliding window, is often used instead of any single SNP [120]. Genotyping-by-sequencing (GBS)-based mapping was successfully first demonstrated in rice [120]. GBS can greatly reduce the financial cost and labor required for linkage mapping compared to traditional mapping using PCR-based markers [120,121].

GBS have thus far been used to map many important production-related loci in crops. Nevertheless, up till now, there have been limited successful cases describing the mapping of genes from wild relatives in grain legumes. GBS of a unique RI population of *G. max* × *G. soja* has successfully identified a major QTL conferring salt tolerance in wild soybean [66]. Combined with the association study of resequencing consensus of 20 unrelated germplasms and the comparison of de novo genomes of cultivated soybean and wild soybean, the authors have identified the causal gene for salt tolerance in the wild parent to be a gene encoding a cation/proton exchanger (*GmCHX1*) [66]. Similarly, a recent potential multidrug and toxic compound extrusion (MATE) transporter has been identified to be associated with the total contents of antioxidants, phenolics, and flavonoids in soybean seeds. This common genomic region for the three groups of compounds can explain up to 64% of the phenotypic variance under field conditions [9] (Table 2). Another trait studied using GBS is the resistance to sclerotinia stem rot disease in soybean. After genotyping 101 soybean lines with different levels of resistance, the researchers found three major QTLs, distributed on chromosomes Gm03, Gm08 and Gm20 [122] (Table 2). An excellent approach using a big soybean collection including wild germplasms and whole-genome resequencing allowed researchers to identify important genes related to domestication and crop improvement, making use not only of the new sequencing technologies but also of the information generated during many years of characterizing QTLs and genes in soybean [80]. Soybean root architecture [123] and total fresh weight [124] seem to be clearly associated with a specific region in the soybean genome. Same results have been generated by two independent groups using different wild and cultivated soybean populations and approaches (Table 2).

Populations and mapping using cultivated × wild or landrace of common beans have been developed to dissect agronomic traits such as white mold resistance (NILs and BC) [125,126] as well as seed weight, seed size, days to flowering, yield, plant height and concentration of minerals such as Zn and Fe in seeds [127,128] (Table 2). The recently available information on the common bean genome (Table 1) together with the re-sequenced genome, transcriptome and methylome [41,72,129,130] will allow the scientific community to speed up the dissection of important traits for this grain legume using GBS approaches [131,132,133].

In the case of chickpea, the role of wild species for mapping and crop improvement has been extensively reviewed [160,161] and huge efforts have been made to develop different mapping populations including wild chickpea and landraces (Table 2) for traits such as flowering time, 100-seed weight, pod and branch number per plant, plant hairiness, seed yield per plant, etc. [137,138,139,140,141]. During the past several years, important articles reporting on the genetics and genomics of chickpea have deepened our understanding of this legume and contributed to the possibility of developing new approaches to improve this crop by characterizing the genetic diversity in the wild species [68,69,70,71,83,140,162,163,164,165].

The developing of mapping populations using wild or diploid ancestors of peanut has presented a special challenge, given the ploidy difference and sexual incompatibility between wild and cultivated peanut. Therefore generating the mapping populations sometimes involves the development of wild synthetic allotetraploids [166]. Still, there are five examples of using wild relatives to successfully map important genetic regions controlling root-knot nematode resistance, drought- and agronomic/domestication-related traits, flowering precocity, seed and pod numbers, pod length and size, pod maturity time, height of the main stem, plant spread, flower color and late leaf spot resistance [142,143,144,146,167] (Table 2). Janila et al. (2016) [29] also summarized up to the present, the history and perspectives of genetics and genomics-assisted breeding (Appendix A) in peanut, highlighting the importance of wild relatives as a source of novel alleles.

In the case of pea, an RIL population between the wild relative, *Pisum sativum* subsp. *Syriacum*, and a cultivated line has been developed, revealing six QTLs related to *Mycosphaerella pinodes* resistance (Table 2). Interesting QTLs related with domestication features have been described using five populations generated by crossing lines representing different stages of domestication (e.g., wild, landrace, etc.) (Table 2). Pea landraces tolerant of abiotic stresses such as frost, drought and high temperature have been identified with great potential as germplasms for breeding target [168]. Although there have not been many studies on QTL mapping using wild relatives together with traditional or new GBS-derived markers up to now, the use of GBS is starting to rise for pea [101]. For instance, a study using RILs from cultivated lines has yielded high-density and high-quality SNP markers with great potentials [169]. A recent review summarized the current status of genomic tools in pea breeding programs [102], which could be applied to better explore wild germplasm of pea.

As a globally popular food crop, lentil has attracted more and more attention from researchers. The wild lentil relative, *Lens ervoides* “Brign”, was crossed with the cultivated lentil line, “Eston”, and the resulting RIL population was then phenotyped for 23 important and complex traits including anthracnose resistance. There is great potential for this population to be genotyped using new technologies and further explored [153] (Table 2). In addition, next-generation sequencing of both the wild and cultivated lentils revealed a large collection of SNPs and improved the genotyping platform for the mapping of the *L. culinaris* genome [170].

Pigeonpea is a cross-pollinated diploid crop. Great efforts have been made by the International Crops Research Institute for the Semi-Arid Tropics (ICRISAT) over the years to increase productivity. Diverse breeding strategies are continuously being developed for pigeonpea improvement [171]. The developments of cytoplasmic male sterile lines for hybrid breeding have systematically and steadily been generating the most promising materials available [151,172,173,174]. Incompatible crossing barriers between cultivated pigeonpea and its wild relatives have not hindered its improvement [175,176]. A number of interesting examples show the improvements on this grain legume using features in alien germplasms, such as abiotic stress (salinity) tolerance and resistance to biotic stress (fusarium wilt, phytophthora blight and cyst nematode), genetic dwarfs, high protein content and special nutritional value, cleistogamy, male sterility lines, etc. [149,150,151,152,175,177] (Table 2). A recent excellent review summarized the current status of genomics-assisted breeding in pigeonpea [175] after the release of the first draft genome.

Cowpea is a readily self-pollinating crop. Using cultivated cowpea as well as wild relatives and landraces, an important number of mapping populations have been developed during the past 30 years. Close cross-compatible relatives have been explored as genetic reservoirs and parent donors of important agronomic traits [178,179,180]. The available high-marker-density linkage map using synteny with other legumes have increased the possibilities to search for regions and genes associated with agronomically important traits and a series of databases strongly facilitate cowpea breeding [181]. Most of them have been developed using cultivated cowpea genotypes, demonstrating a great potential for increasing the genetic diversity of this cultivated pulse [178]. A set of traits, such as floral scent compounds, seed size, pod fiber layer thickness, seed weight, time of flower opening, days to flower, have also been improved using genetic resources from wild relatives [154,155,156] (Table 2). Some population approaches evaluating pod length, pod tenderness and domestication related traits have also been developed in yardlong bean (*Vigna unguiculata* [L.] Walp. ssp. *unguiculata* cv.-gr. *sesquipedalis*), which, interestingly, has evolved from cowpea by divergence domestication in Asia [157,158,159].

In the case of lupins, mapping studies have been done, either through traditional molecular markers or high-throughput sequencing techniques, to complement breeding programs for different species within the complex [75,99,182,183,184,185]. However, total or low-frequency crossing barriers between species of *Lupinus* make the scenario of interspecific gene transfer too complicated to improve specific agronomic traits [100]. Some examples of interspecific crosses have been reported but the situation is far too complicated to be a useful tool for lupin breeding [186].

## 6. Use of Genetic Diversity in Wild Relatives to Improve Grain Legume Performance

Taking advantage of the genetic diversity of wild relatives of crop plants to improve the performance of crops is not a novel concept, but it is worth noting that, for grain legumes, it is often difficult to trace back the breeding history and the real impact on crop production. In contrast, the record-keeping on other major crops seems to be a little more comprehensive [187]. It seems that grain legumes, especially those cultivated in more limited geographical areas, have been systematically neglected [13].

The advent of genomics and genomics-assisted breeding have expanded our understanding of complex traits, allowing us to dissect the genetic bases of traditional and new important agronomical traits, not only to increase productivity or adapt to climate change, but also to develop alternative food production systems tailored to poor areas and small farms to grow more and better food.

The use of genomics-assisted breeding using wild relatives, particularly in grain legumes, should be intensified. The skepticism of plant breeders to make use of wild and exotic plant genetic resources due to associated linkage drag is gradually overcome because the exploration of expanded gene pools is providing us with unprecedented opportunities to discover major genes controlling important traits. Once the major genes or genomic regions have been characterized, it would be worthwhile to move forward the introgression from the cultivated populations and even consider transgenesis, genome editing or the less controversial mutagenesis. Scientific developments and the knowledge generated thereof using wild relatives are revolutionizing our understanding of biological processes. However, it has been cautioned that the improvement of specific traits using wild relatives masks the true potential of genetic diversity in the wild relatives for breeding [188]. Clearly, the potential with using wild relatives goes beyond crop improvement itself. It is a promising scenario where international collaborations shall arise and deepen in order to contribute to increased food production, environmental sustainability and better quality of life for future generations.

In Table 2, we summarize published works on genes or genomic regions associated with a wide range of important traits from reproductive features to interactions with microorganisms, by mining the genetic resources in wild relatives for different types of population development. In this work, we have compiled the most up-to-date information on population approaches using wild relatives of the nine grain legumes. Only a few of these major works have been performed using available genotyping technology by deep sequencing, and even fewer of them explored the diversity of genomic information for the characterized regions. Finally, the various grain legume populations already generated and summarized in this review are in themselves great resources for further exploration and deeper genomic and genetic analyses, especially with respect to the -omics information on their wild versus cultivated germplasms.

## 7. Conclusions and Perspectives

Although we have long recognized the value of wild relatives in the past, there always seem to be a repeated pattern of recognizing their potential importance following periods of calamities in food production. An example of great interests in wild crop relatives after the Second World War [189], and a second, after the devastating losses in maize production occurred in USA in 1970. Both periods led to significant advances in the collection and evaluation of wild materials culminating in the formation of the International Plant Genetic Resources Institute (today operating under the name, Biodiversity International) [4].

The contribution of wild relatives to modern agriculture, helping to increase yield, quality and disease resistance, is significant. Joint efforts from plant breeders and collaborative institutions across the world have contributed to the examples of success. The linkage drag and reproductive barriers are a hard hurdle to overcome and the contributions made thus far to dealing with this issue should be especially recognized. However, the genetic erosion of the most important crops remains problematic, particularly when we are facing more frequent periods of flooding, drought and/or diseases with global warming, coupled with intensive agricultural systems. In this regard, the use of wild relatives may be the solution.

Tanksley and McCouch [4] proposed the paradigm shift from “looking at the phenotype” to “looking at the gene” during the screening of exotic germplasm, shifting away from selecting potential parents based in phenotype to evaluate directly the presence of novel genes. The examples given in this review are some of the biggest successes and plant breeders have continued working on this idea. With the emergence of next-generation sequencing techniques, several studies using wild relatives have been published, generating important information about genetic diversity, population structure, gene expression, methylation patterns, adaptation mechanisms, etc. Our abilities to characterize and understand the genetic variability are the basis for this paradigm shift, based on examining the genetic composition rather than the phenotype. We face a big challenge related to the scattering of characterized genetic diversity information generated by researchers around the world and deposited in various genetic resource centers. It is important to emphasize the importance of characterizing not only the traditional morpho-agronomic traits but also those more complex traits that are crucial for enhancing the potential to adapt to future climate scenarios. Characterization and accessibility to core and mini core collections of pulses diversity is a fundamental requirement for any breeding approach [190,191]. The organization and availability of this vast amount of new information must be made a worldwide priority in order to facilitate the use of these genetic resources [192,193]. In an excellent review for the International Year of Pulses, the number of accessions and locations of grain legume collections around the world have been collated [13]. The discovery of new wild alleles controlling specific traits in specific crops should be more easily facilitated with the centralization of genomics information into big databases that contain important information such as specific gene expressions in different tissues, at different developmental stages or under different stress conditions [194].

One major limitation in the utilization of crop wild relatives in breeding programs is due to major gaps in the genetic diversity of “gene pools”. The availability of crop wild relatives could be hampered by many factors, such as loss of natural habitats. “Gap analysis” is a tool to assess genetic conservation and to formulate conservation strategies by prioritizing among taxa containing gaps due to sampling, geographic and environmental factors. The power of this tool was demonstrated by a case study of *Phaseolus* gene pool [195,196].

A big international effort is underway with the aim to adapt agriculture to climate change, which includes collecting, protecting and preparing crop wild relatives. Several pulses are among the major targets: common bean, adzuki bean, chickpea, cowpea, faba bean, groundnut, lentil, lima bean, mung bean, pea, pigeonpea, soybean, urd bean and vetch [197,198]. The information generated and systematized from this project certainly will be a unique source of information and materials facing the current and futures challenges for agriculture in the context of crop wild relatives use.

## Figures and Tables

**Table 1:** De novo whole-genome sequencing results for various grain legumes.

Crop	Genus	Species	Germplasm	Assembled Genome Size (Mb)	Predicted Genome Size (Mb)	Available Database(s) *	Reference
Soybean	*Glycine*	*max*	Cultivated (William 82)	950	1115	1, 2	[65]
Soybean	*Glycine*	*soja*	Wild	868	1170		[66]
Soybean	*Glycine*	*soja*	Wild	813–985	889–1118		[67]
Peanut	*Arachis*	*duranensis*	Wild	1211	1250	3	[28]
Peanut	*Arachis*	*ipaensis*	Wild	1512	1560		[28]
Pea	*Pisum*	*sativum*	N/D	N/D	4685 #	4, 5	[13]
Chickpea	*Cicer*	*arietinum*	Cultivated (*desi*-type)	520	740	6, 4, 7	[68]
Chickpea	*Cicer*	*arietinum*	Cultivated (*kabuli*-type)	532	738		[69]
Chickpea	*Cicer*	*arietinum*	Cultivated (*desi*-type)	511	740		[70]
Chickpea	*Cicer*	*reticulatum*	Wild	416	817		[71]
Common bean	*Phaseolus*	*vulgaris*	Landrace	473	587	6, 4, 8, 2	[41]
Common bean	*Phaseolus*	*vulgaris*	Breeding lines	550	587		[72]
Lentil	*Lens*	*culinaris*	N/D	N/D	4032 #	4	[13]
Cowpea	*Vigna*	*unguiculata*	Cultivated (IT97K-499-35)	Not complete	620	9	[73]
Lupin	*Lupinus*	*angustifolius*	Cultivated (Tanjil)	609	951	6, 10, 11	[74]
Lupin	*Lupinus*	*angustifolius*	Cultivated (Tanjil)	598	1153		[75]
Pigeonpea	*Cajanus*	*cajan*	Cultivated (Asha)	606	833	6	[76]
Pigeonpea	*Cajanus*	*cajan*	Cultivated (Asha)	511	858		[77]

* Major online databases for de novo genome information: N/D no data available; # The predicted genome size is extracted from [13]: 1, http://soybase.org; 2, https://phytozome.jgi.doe.gov/pz/portal.html; 3, http://peanutbase.org/home; 4, http://knowpulse.usask.ca/portal/; 5, https://urgi.versailles.inra.fr/Data/Genome/Genome-data-access; 6, http://legumeinfo.org/genomes; 7, http://nipgr.res.in/CGAP/home.php; 8, http://phaseolusgenes.bioinformatics.ucdavis.edu/; 9, http://cowpeagenomics.med.virginia.edu/CGKB; 10, http://www.lupinexpress.org/node/6; 11, http://www.ncbi.nlm.nih.gov/bioproject/PRJNA179231.

**Table 2:** Trait-related QTLs/genes in grain legumes characterized using wild relatives.

Grain Legume	Trait/s	Population Strategy	Genotyping/Mapping Strategy	QTL/Gene	Reference
Soybean	Salt tolerance, seed anthocyanin content, pod and seed number per plant, growth period, seed coat color, pod color, trailing growth, leaf length/width ratio, nodule number per plant with cultivated incompatible rhizobia strain	RILs (cultivated × wild)	GBS-WGR	CHX cation anti-transporter for salt tolerance, QTLs for the others traits	[66]
Soybean	Nodule fresh weight per plant, root fresh weight, total plant fresh weight and ureides (μmol per plant)	RILs (cultivated × wild)	GBS-WGR	QTLs	[124]
Soybean	Antioxidants, phenolics, and flavonoids in seeds	RILs (cultivated × wild)	GBS-WGR	MATE transporters	[9]
Soybean	Sclerotinia stem rot resistance	101 lines	GBS-association mapping	QTL	[122]
Soybean	Root traits (tap root length and lateral root number) and shoot length	BC_2_F_5_ (cultivated × wild)	SSR and SNP markers	QTLs	[123]
Soybean	Oil content, flower color, seed coat color, pubescence form and reported domestication-related QTLs	302 wild and cultivated accessions	GBS-association mapping	GWAS signals associated	[80]
Soybean	Yield, height and maturity	BC_2_F_4_ (cultivated × wild)	SSR markers	QTLs	[134]
Soybean	Soybean cyst nematode resistance	RILs (cultivated × wild)	SSR markers	QTLs	[135]
Soybean	Soybean cyst nematode resistance	235 wild soybean accessions	GBS-GWAS	QTLs	[136]
Common bean	White mold resistance	NILs source of resistance from Andean genotype Jatu Rong	InDel, SCAR, SNP and phaseolin markers	QTLs	[126]
Common bean	Seed weight, seed size, days to flowering, yield, plant height	BC_2_F_3:5_ (cultivated × wild)	Microsatellite, SCAR, and phaseolin markers	QTLs	[127]
Common bean	White mold resistance	BC_2_F_3_ (cultivated × wild) BC_1_F_4__:5_ (landrace × cultivated)	SSR, SRAP, TRAP markers	QLTs	[125]
Common bean	Seed weight, seed mineral accumulation: iron concentration (ppm), zinc concentration (ppm), iron content (mg/seed), zinc content (mg/seed)	BC_2_F_3:5_ (cultivated × wild)	Microsatellite markers	QTLs	[128] and cited herein
Chickpea	Seed coat color	Germplasm collection (93 cultivated *desi* and *kabuli* and 79 wild) and RILs (landrace × landrace)	GBS-WGR-association and QTL mapping	MATE transporter	[137]
Chickpea	Flowering time	Germplasm collection (92 cultivated *desi* and *kabuli* including beaded, landraces and wild)	Genome-wide GBS- and candidate gene-based genotyping	Eight potential known/candidate flowering time-regulating genes and QTLs	[138]
Chickpea	100-seed weight, pod and branch number/plant and plant hairiness	RILs (cultivated × wild)	SSR and SNP polymorphism marker-based	QTLs and seed weight regulating ABI3VP1 transcription factor	[139]
Chickpea	Pod number and seed yield per plant	Two F_5_ mapping populations (cultivated × wild)	GBS-WGR SNP InDel markers	QTLs	[140,141]
Chickpea	100-seed weight	RILs (cultivated × landrace)	GBS-WGR	QTLs	[137]
Peanut	Root-knot nematodes resistance, drought-related traits and agronomic/domestication traits	RILs (wild × wild)	SNP markers and integrated consensus map from Shirasawa et al. 2013 [142]	QTLs	[143]
Peanut	Water availability, flowering precocity, seed and pod number, length and size, and pod maturity	87 BC_3_F_1_ and 55 BC_2_F_2_ [cultivated × wild amphidiploid (*A. ipaensis* × *A. duranensis*)]	SSR markers	QTLs	[144]
Peanut	Root-knot nematode resistance	BC_4_F_2_ population (cultivated × wilds)	RAPD markers	Resistance associated to markers	[145]
Peanut	Plant growth habit, height of the main stem, plant spread and flower color	Chromosome segment substitution lines (CSSLs). [wild synthetic allotetraploid (*A. ipaensis* × *A. duranensis*) × cultivated]	SSR markers	QTLs	[146]
Peanut	Late leaf spot resistance	F_2_ (*A. duranensis* × *A. stenosperma*)	Microsatellite, AFLP and legume anchor markers	QTLs	[142]
Pea	*Mycosphaerella pinodes* resistance	RILs (cultivated × wild)	RAPD, Sequence-tagged site and expressed sequence tag markers	QTLs	[147]
Pea	Seed weight, root/shoot ratio, flowering response, pod dehiscence, seed dormancy, plant height, basal branching	Set of five recombinant inbred populations: wild × cultivated F_12_; cultivated × wild F_6_; cultivated × primitive BC_1_F_4_; primitive × wild F_4_; cultivated × landrace F_5_	Morphological Markers, allozyme variation and RAPD	QTLs and genes	[148]
Pigeonpea	Cleistogamous line	F_2_ (cultivated × wild)	N/A	Gene	[149]
Pigeonpea	High protein	Cultivated × wild	N/A	Traditional breeding (pedigree method)	[150]
Pigeonpea	Male sterility lines	BC_n_ (cultivated × wild)	N/A	Traditional breeding	[151]
Pigeonpea	Drought tolerance and pod borer insect resistance	F_2_ (wild × cultivated)	Single feature polymorphisms (SFPs)	Genes	[152]
Lentil	Anthracnose resistance, seed yield, biomass, straw yield, seed weight, harvest index, podding ability and stand at maturity	RILs (wild × cultivated)	N/A	N/A potential material for breeding	[153]
Cowpea	Floral scent compounds, seed size, pod fiber layer thickness, seed weight, time of flower opening, days to flower	RILs (cultivated × wild)	SSR	QTLs	[154,155,156]
Yardlong bean (*Vigna unguiculata* ssp. *unguiculata* cv.-gr. sesquipedalis)	Pod length	BC_1_F_1_ (cultivated × wild)	SSR	QTLs	[157]
Yardlong bean	Domestication related traits	BC_1_F_1_ (cultivated × wild)	SSR	QTLs	[158]
Yardlong bean	Pod tenderness	BC_1_F_1_ and F_2_ (cultivated × wild)	SSR	QTLs	[159]

## Abbreviations

QTL	Quantitative trait locus
InDel	Insertion/deletion
CNV	Copy number variation
PAV	Present/absent variation
cM	Centimorgan
A-BC	Advanced backcross population
RIL	Recombinant inbred line
NIL	Near-isogenic line
NAM	Nested association mapping
MAGIC	Multi-parent advanced generation inter-cross
LD	Linkage disequilibrium
GWAS	Genome-wide association studies
SNP	Single nucleotide polymorphism
DArT	Diversity Arrays Technology
RAD	Restriction site-associated DNA marker
RRL	Reduced-representation library
CRoPS	Complexity reduction of polymorphic sequence
GBS	Genotyping-by-sequencing
IBL	Inbred backcross line
MATE	Multidrug and toxic compound extrusion
SRAP	Sequence-related amplified polymorphism
TRAP	Target region amplification polymorphism
SRR	Simple sequence repeat
RAPD	Random amplified polymorphic DNA
AFLP	Amplified fragment length polymorphism

## Appendix A

Gene	A DNA sequence that determines the appearance of hereditary characteristics in living organisms
Allele	Each alternative form of a gene, occupying the same position in each pair of homologous chromosomes
Fst (fixation index)	Measure of population differentiation due to the genetic structure
Phenotype	A set of inherited characteristics that is dependent on both the genes and the environment
Genotype	A set of genes that are characteristic of each organism or individual
QTL (quantitative trait locus)	A locus on the chromosome that is associated with the quantitative variation of a trait
Association mapping	A germplasm-based approach to characterize QTLs or variations by exploiting the historic linkage disequilibrium to associate phenotypes with the underlying genotypes
Joint-linkage association mapping	A family-based approach to characterize QTLs or variations that are shared across families. It differs in power and scope from the characterization of QTLs based on bi-parental populations.
Marker-assisted selection	A set of tools that use gene markers to select, in a precise manner, the plants with the genetic potential to produce the desired trait for breeding
Genomics-assisted breeding	A set of genomics tools (using high-throughput approaches) to select in a targeted manner the plants with the genetic potential to produce the desired trait for breeding
Introgression	The gene flow from one genetic background (individual) to another gene pool (individual) by backcrossing with one of its parent
Backcross	Crossing of offspring lines with one of the original parental line
Linkage drag	Offspring with undesirable genetic background inherited from one of the parental lines
SNP (Single Nucleotide Polymorphism)	Single-nucleotide variations on the DNA sequence within a population or between paired chromosomes

## References

[1] Acquaah G. (2009). Principles of Plant Genetics and Breeding.

[2] FAO How to Feed the World 2050. http://www.fao.org/wsfs/forum2050/wsfs-forum/en/.

[3] Lobell D.B., Schlenker W., Costa-Roberts J. (2011). Climate trends and global crop production since 1980. Science.

[4] Tanksley S.D., McCouch S.R. (1997). Seed banks and molecular maps: Unlocking genetic potential from the wild. Science.

[5] Dwivedi S.L., Upadhyaya H.D., Stalker H.T., Blair M.W., Bertioli D.J., Nielen S., Ortiz R. (2008). Enhancing crop gene pools with beneficial traits using wild relatives. Plant Breed. Rev..

[6] Harlan J.R. (1976). Genetic resources in wild relatives of crops. Crop Sci..

[7] McCouch S. (2004). Diversifying selection in plant breeding. PLoS Biol..

[8] Huang X.H., Han B. (2014). Natural variations and genome-wide association studies in crop plants. Annu. Rev. Plant Biol..

[9] Li M.-W., Muñoz N.B., Wong C.-F., Wong F.-L., Wong K.-S., Wong J.W.-H., Qi X., Li K.-P., Ng M.-S., Lam H.-M. (2016). QTLs regulating the contents of antioxidants, phenolics, and flavonoids in soybean seeds share a common genomic region. Front. Plant Sci..

[10] Asif M., Rooney L.W., Ali R., Riaz M.N. (2013). Application and opportunities of pulses in food system: A review. Crit. Rev. Food Sci. Nutr..

[11] Singh R.J., Nelson R.L. (2014). Methodology for creating alloplasmic soybean lines by using *Glycine tomentella* as a maternal parent. Plant Breed..

[12] Zamir D. (2001). Improving plant breeding with exotic genetic libraries. Nat. Rev. Genet..

[13] Foyer C.H., Lam H.-M., Nguyen H.T., Siddique K.H., Varshney R.K., Colmer T.D., Cowling W., Bramley H., Mori T.A., Hodgson J.M. (2016). Neglecting legumes has compromised human health and sustainable food production. Nat. Plants.

[14] Smýkal P., Coyne C.J., Ambrose M.J., Maxted N., Schaefer H., Blair M.W., Berger J., Greene S.L., Nelson M.N., Besharat N. (2015). Legume crops phylogeny and genetic diversity for science and breeding. Crit. Rev. Plant Sci..

[15] Moose S.P., Mumm R.H. (2008). Molecular plant breeding as the foundation for 21st century crop improvement. Plant Physiol..

[16] Nutritional Benefits of Pulses. http://www.fao.org/fileadmin/user_upload/pulses-2016/docs/factsheets/Nutrition_EN_PRINT.pdf.

[17] Kislev M.E., Bar-Yosef O. (1988). The legumes: The earliest domesticated plants in the near east?. Curr. Anthropol..

[18] Zohary D., Hopf M. (1973). Domestication of pulses in the old world: Legumes were companions of wheat and barley when agriculture began in the Near East. Science.

[19] Harlan J.R., de Wet J.M. (1971). Toward a rational classification of cultivated plants. Taxon.

[20] Hymowitz T. (1970). On the domestication of the soybean. Econ. Bot..

[21] Soybeans (*Glycine max*)—The Plant History of the Marvelous Soybean. http://archaeology.about.com/od/Domesticated-Plants/fl/Soybeans-Glycine-max-The-Plant-History-of-the-Marvelous-Soybean.htm.

[22] Smith K. Soybeans-History and Future. http://www.soymeal.org/FactSheets/HistorySoybeanUse.pdf.

[23] Singh A., Simpson C. (1994). Biosystematics and genetic resources. The Groundnut Crop.

[24] Bonavia D. (1982). Los Gavilanes: Mar, Desierto y oásis en la Historia del Hombre: Precerámico Peruano.

[25] Krapovickas A.G. (1994). WC Taxonomía del género arachis (Leguminosae). Bonplandia.

[26] Dillehay T.D., Rossen J., Andres T.C., Williams D.E. (2007). Preceramic adoption of peanut, squash, and cotton in Northern Peru. Science.

[27] Grabiele M., Chalup L., Robledo G., Seijo G. (2012). Genetic and geographic origin of domesticated peanut as evidenced by 5S rDNA and chloroplast DNA sequences. Plant Syst. Evol..

[28] Bertioli D.J., Cannon S.B., Froenicke L., Huang G., Farmer A.D., Cannon E.K.S., Liu X., Gao D., Clevenger J., Dash S. (2016). The genome sequences of *Arachis duranensis* and *Arachis ipaensis*, the diploid ancestors of cultivated peanut. Nat. Genet..

[29] Janila P., Variath M.T., Pandey M.K., Desmae H., Motagi B.N., Okori P., Manohar S.S., Rathnakumar A.L., Radhakrishnan T., Liao B. (2016). Genomic tools in groundnut breeding program: Status and perspectives. Front. Plant Sci..

[30] Daniel Z., Maria H. (2000). Domestication of Plants in the Old World.

[31] Ambrose M. (1995). From Near East center of origin, the prized pea migrates throughout world. Divers. Arlingt. Then Wash..

[32] Smýkal P., Aubert G., Burstin J., Coyne C.J., Ellis N.T., Flavell A.J., Ford R., Hýbl M., Macas J., Neumann P. (2012). Pea (*Pisum sativum* L.) in the genomic era. Agronomy.

[33] Pea (*Pisum sativum* L.) Domestication-the History of Peas and Humans. http://archaeology.about.com/od/Domesticated-Plants/fl/Pea-Pisum-sativum-L-Domestication-The-History-of-Peas-and-Humans.htm.

[34] Smýkal P., Kenicer G., Flavell A.J., Corander J., Kosterin O., Redden R.J., Ford R., Coyne C.J., Maxted N., Ambrose M.J. (2011). Phylogeny, phylogeography and genetic diversity of the *Pisum genus*. Plant Genet. Resour..

[35] Kerem Z., Lev-Yadun S., Gopher A., Weinberg P., Abbo S. (2007). Chickpea domestication in the neolithic levant through the nutritional perspective. J. Archaeol. Sci..

[36] Shahal A., Inbar Z., Efrat S., Simcha L.-Y., Zohar K., Avi G. (2008). Wild lentil and chickpea harvest in Israel: Bearing on the origins of Near Eastern farming. J. Archaeol. Sci..

[37] Warkentin T., Banniza S., Vandenberg A. (2005). CDC frontier *kabuli* chickpea. Can. J. Plant Sci..

[38] Delgado-Salinas A., Bibler R., Lavin M. (2006). Phylogeny of the genus *Phaseolus* (Leguminosae): A recent diversification in an ancient landscape. Syst. Bot..

[39] Delgado-Salinas A., Turley T., Richman A., Lavin M. (1999). Phylogenetic analysis of the cultivated and wild species of *Phaseolus* (Fabaceae). Syst. Bot..

[40] Freytag G.F., Debouck D.G. (1996). *Phaseolus costaricensis*, a new wild bean species (Phaseolinae, Leguminosae) from Costa Rica and Panama, Central America. Novon.

[41] Schmutz J., McClean P.E., Mamidi S., Wu G.A., Cannon S.B., Grimwood J., Jenkins J., Shu S., Song Q., Chavarro C. (2014). A reference genome for common bean and genome-wide analysis of dual domestications. Nat. Genet..

[42] Bitocchi E., Nanni L., Bellucci E., Rossi M., Giardini A., Zeuli P.S., Logozzo G., Stougaard J., McClean P., Attene G. (2012). Mesoamerican origin of the common bean (*Phaseolus vulgaris* L.) is revealed by sequence data. Proc. Natl. Acad. Sci. USA.

[43] Bellucci E., Bitocchi E., Rau D., Rodriguez M., Biagetti E., Giardini A., Attene G., Nanni L., Papa R. (2014). Genomics of origin, domestication and evolution of *Phaseolus vulgaris*. Genomics of Plant Genetic Resources.

[44] Ljuština M., Mikić A. (2010). Archaeological evidence for the domestication of lentil (*Lens culinaris*) and its distribution in Europe. J. Lentil Res..

[45] Sonnante G., Hammer K., Pignone D. (2009). From the cradle of agriculture a handful of lentils: History of domestication. Rend. Lincei.

[46] D’Andrea A.C., Kahlheber S., Logan A.L., Watson D.J. (2007). Early domesticated cowpea (*Vigna unguiculata*) from Central Ghana. Antiquity.

[47] Singh B. (1997). Advances in Cowpea Research.

[48] Perrino P., Laghetti G., Zeuli P.S., Monti L. (1993). Diversification of cowpea in the mediterranean and other centres of cultivation. Genet. Resour. Crop Evol..

[49] Kurlovich B.S. (2002). Lupins: Geography, Classification, Genetic Resources and Breeding.

[50] Fuller D., Korisettar R., Venkatasubbaiah P., Jones M.K. (2004). Early plant domestications in Southern India: Some preliminary archaeobotanical results. Veg. Hist. Archaeobot..

[51] Fuller D.Q., Harvey E.L. (2006). The archaeobotany of indian pulses: Identification, processing and evidence for cultivation. Environ. Archaeol..

[52] Gepts P., Papa R. (2002). Evolution during domestication. eLS.

[53] Fuller D.Q., Denham T., Arroyo-Kalin M., Lucas L., Stevens C.J., Qin L., Allaby R.G., Purugganan M.D. (2014). Convergent evolution and parallelism in plant domestication revealed by an expanding archaeological record. Proc. Natl. Acad. Sci. USA.

[54] Abbo S., van-Oss R.P., Gopher A., Saranga Y., Ofner I., Peleg Z. (2014). Plant domestication versus crop evolution: A conceptual framework for cereals and grain legumes. Trends Plant Sci..

[55] Fuller D.Q. (2007). Contrasting patterns in crop domestication and domestication rates: Recent archaeobotanical insights from the old world. Ann. Bot..

[56] Kluyver T.A., Charles M., Jones G., Rees M., Osborne C.P. (2013). Did greater burial depth increase the seed size of domesticated legumes?. J. Exp. Bot..

[57] Abbo S., Lev-Yadun S., Gopher A. (2014). The “human mind” as a common denominator in plant domestication. J. Exp. Bot..

[58] Zeder M.A. (2011). The origins of agriculture in the Near East. Curr. Anthropol..

[59] Larson G., Piperno D.R., Allaby R.G., Purugganan M.D., Andersson L., Arroyo-Kalin M., Barton L., Vigueira C.C., Denham T., Dobney K. (2014). Current perspectives and the future of domestication studies. Proc. Natl. Acad. Sci. USA.

[60] Meyer R.S., DuVal A.E., Jensen H.R. (2012). Patterns and processes in crop domestication: An historical review and quantitative analysis of 203 global food crops. New Phytol..

[61] Kaul S., Koo H.L., Jenkins J., Rizzo M., Rooney T., Tallon L.J., Feldblyum T., Nierman W., Benito M.I., Lin X.Y. (2000). Analysis of the genome sequence of the flowering plant *Arabidopsis thaliana*. Nature.

[62] International Rice Genome Sequencing Project (2005). The map-based sequence of the rice genome. Nature.

[63] Yu J., Hu S., Wang J., Wong G.K., Li S., Liu B., Deng Y., Dai L., Zhou Y., Zhang X. (2002). A draft sequence of the rice genome (*Oryza sativa* L. ssp. *Indica*). Science.

[64] Hayden E.C. (2014). Technology: The $1000 genome. Nature.

[65] Schmutz J., Cannon S.B., Schlueter J., Ma J., Mitros T., Nelson W., Hyten D.L., Song Q., Thelen J.J., Cheng J. (2010). Genome sequence of the palaeopolyploid soybean. Nature.

[66] Qi X., Li M.-W., Xie M., Liu X., Ni M., Shao G., Song C., Yim A.K.-Y., Tao Y., Wong F.-L. (2014). Identification of a novel salt tolerance gene in wild soybean by whole-genome sequencing. Nat. Commun..

[67] Li Y.-H., Zhou G., Ma J., Jiang W., Jin L.-G., Zhang Z., Guo Y., Zhang J., Sui Y., Zheng L. (2014). De novo assembly of soybean wild relatives for pan-genome analysis of diversity and agronomic traits. Nat. Biotechnol..

[68] Jain M., Misra G., Patel R.K., Priya P., Jhanwar S., Khan A.W., Shah N., Singh V.K., Garg R., Jeena G. (2013). A draft genome sequence of the pulse crop chickpea (*Cicer arietinum* L.). Plant J..

[69] Varshney R.K., Song C., Saxena R.K., Azam S., Yu S., Sharpe A.G., Cannon S., Baek J., Rosen B.D., Tar’an B. (2013). Draft genome sequence of chickpea (*Cicer arietinum*) provides a resource for trait improvement. Nat. Biotechnol..

[70] Parween S., Nawaz K., Roy R., Pole A.K., Suresh B.V., Misra G., Jain M., Yadav G., Parida S.K., Tyagi A.K. (2015). An advanced draft genome assembly of a *desi* type chickpea (*Cicer arietinum* L.). Sci. Rep..

[71] Gupta S., Nawaz K., Parween S., Roy R., Sahu K., Pole A.K., Khandal H., Srivastava R., Parida S.K., Chattopadhyay D. (2016). Draft genome sequence of *Cicer reticulatum* L., the wild progenitor of chickpea provides a resource for agronomic trait improvement. DNA Res..

[72] Vlasova A., Capella-Gutiérrez S., Rendón-Anaya M., Hernández-Oñate M., Minoche A.E., Erb I., Câmara F., Prieto-Barja P., Corvelo A., Sanseverino W. (2016). Genome and transcriptome analysis of the Mesoamerican common bean and the role of gene duplications in establishing tissue and temporal specialization of genes. Genome Biol..

[73] Timko M.P., Rushton P.J., Laudeman T.W., Bokowiec M.T., Chipumuro E., Cheung F., Town C.D., Chen X. (2008). Sequencing and analysis of the gene-rich space of cowpea. BMC Genom..

[74] Hane J.K., Ming Y., Kamphuis L.G., Nelson M.N., Garg G., Atkins C.A., Bayer P.E., Bravo A., Bringans S., Cannon S. (2016). A comprehensive draft genome sequence for lupin (*Lupinus angustifolius*), an emerging health food: Insights into plant-microbe interactions and legume evolution. Plant Biotechnol. J..

[75] Yang H., Tao Y., Zheng Z., Zhang Q., Zhou G., Sweetingham M.W., Howieson J.G., Li C. (2013). Draft genome sequence, and a sequence-defined genetic linkage map of the legume crop species *Lupinus angustifolius* L.. PLoS ONE.

[76] Varshney R.K., Chen W., Li Y., Bharti A.K., Saxena R.K., Schlueter J.A., Donoghue M.T., Azam S., Fan G., Whaley A.M. (2012). Draft genome sequence of pigeonpea (*Cajanus cajan*), an orphan legume crop of resource-poor farmers. Nat. Biotechnol..

[77] Singh N.K., Gupta D.K., Jayaswal P.K., Mahato A.K., Dutta S., Singh S., Bhutani S., Dogra V., Singh B.P., Kumawat G. (2012). The first draft of the pigeonpea genome sequence. J. Plant Biochem. Biotechnol..

[78] Lam H.M., Xu X., Liu X., Chen W., Yang G., Wong F.L., Li M.W., He W., Qin N., Wang B. (2010). Resequencing of 31 wild and cultivated soybean genomes identifies patterns of genetic diversity and selection. Nat. Genet..

[79] Chung W.H., Jeong N., Kim J., Lee W.K., Lee Y.G., Lee S.H., Yoon W., Kim J.H., Choi I.Y., Choi H.K. (2014). Population structure and domestication revealed by high-depth resequencing of korean cultivated and wild soybean genomes. DNA Res..

[80] Zhou Z., Jiang Y., Wang Z., Gou Z., Lyu J., Li W., Yu Y., Shu L., Zhao Y., Ma Y. (2015). Resequencing 302 wild and cultivated accessions identifies genes related to domestication and improvement in soybean. Nat. Biotechnol..

[81] Rodriguez M., Rau D., Bitocchi E., Bellucci E., Biagetti E., Carboni A., Gepts P., Nanni L., Papa R., Attene G. (2016). Landscape genetics, adaptive diversity and population structure in *Phaseolus vulgaris*. New Phytol..

[82] Moreno M.-T., Cubero J. (1978). Variation in *Cicer arietinum* L.. Euphytica.

[83] Bajaj D., Das S., Badoni S., Kumar V., Singh M., Bansal K.C., Tyagi A.K., Parida S.K. (2015). Genome-wide high-throughput SNP discovery and genotyping for understanding natural (functional) allelic diversity and domestication patterns in wild chickpea. Sci. Rep..

[84] Kochert G., Halward T., Branch W., Simpson C. (1991). RFLP variability in peanut (*Arachis hypogaea* L.) cultivars and wild species. Theor. Appl. Genet..

[85] Varshney R., Penmetsa R., Dutta S., Kulwal P., Saxena R., Datta S., Sharma T., Rosen B., Carrasquilla-Garcia N., Farmer A. (2010). Pigeonpea genomics initiative (PGI): An international effort to improve crop productivity of pigeonpea (*Cajanus cajan* L.). Mol. Breed..

[86] Kassa M.T., Penmetsa R.V., Carrasquilla-Garcia N., Sarma B.K., Datta S., Upadhyaya H.D., Varshney R.K., von Wettberg E.J., Cook D.R. (2012). Genetic patterns of domestication in pigeonpea (*Cajanus cajan* (L.) Millsp.) and wild cajanus relatives. PLoS ONE.

[87] Lentil Genome Pre-Release. http://knowpulse.usask.ca/portal/lentil-genome.

[88] Plant and Fungi Data Integration. https://urgi.versailles.inra.fr/Data/Genome/Genome-data-access.

[89] The Cowpea Genomics Initiative. http://cowpeagenomics.med.virginia.edu/.

[90] Simon M., Benko-Iseppon A.-M., Resende L., Winter P., Kahl G. (2007). Genetic diversity and phylogenetic relationships in *Vigna* Savi germplasm revealed by DNA amplification fingerprinting. Genome.

[91] Nkongolo K. (2003). Genetic characterization of malawian cowpea (*Vigna unguiculata* (L.) walp) landraces: Diversity and gene flow among accessions. Euphytica.

[92] Ghalmi N., Malice M., Jacquemin J.-M., Ounane S.-M., Mekliche L., Baudoin J.-P. (2010). Morphological and molecular diversity within algerian cowpea (*Vigna unguiculata* (L.) Walp.) landraces. Genet. Resour. Crop Evol..

[93] Wang M.L., Barkley N.A., Gillaspie G.A., Pederson G.A. (2008). Phylogenetic relationships and genetic diversity of the USDA *Vigna* germplasm collection revealed by gene-derived markers and sequencing. Genet. Res..

[94] Wamalwa E.N., Muoma J., Wekesa C. (2016). Genetic diversity of cowpea (*Vigna unguiculata* (L.) Walp.) accession in Kenya gene bank based on simple sequence repeat markers. Int. J. Genom..

[95] Xiong H., Shi A., Mou B., Qin J., Motes D., Lu W., Ma J., Weng Y., Yang W., Wu D. (2016). Genetic diversity and population structure of cowpea (*Vigna unguiculata* L. Walp). PLoS ONE.

[96] Coulibaly S., Pasquet R., Papa R., Gepts P. (2002). AFLP analysis of the phenetic organization and genetic diversity of *Vigna unguiculata* L. Walp. Reveals extensive gene flow between wild and domesticated types. Theor. Appl. Genet..

[97] Michelle C.-A. (2016). Análisis de la Variabilidad Genética entre treinta accesiones de tarwi (*Lupinus mutabilis* Sweet) usando marcadores moleculares ISSR. Sci. Agropecu..

[98] Atchison G.W., Nevado B., Eastwood R.J., Contreras-Ortiz N., Reynel C., Madriñán S., Filatov D.A., Hughes C.E. (2016). Lost crops of the Incas: Origins of domestication of the Andean pulse crop tarwi, *Lupinus mutabilis*. Am. J. Bot..

[99] Iqbal M.J., Mamidi S., Ahsan R., Kianian S.F., Coyne C.J., Hamama A.A., Narina S.S., Bhardwaj H.L. (2012). Population structure and linkage disequilibrium in *Lupinus albus* L. Germplasm and its implication for association mapping. Theor. Appl. Genet..

[100] Wolko B., Clements J.C., Naganowska B., Nelson M.N., Yang H., Kole C. (2011). Wild Crop Relatives: Genomic and Breeding Resources. Legume Crops and Forages.

[101] Sudheesh S., Sawbridge T.I., Cogan N.O., Kennedy P., Forster J.W., Kaur S. (2015). De novo assembly and characterisation of the field pea transcriptome using RNA-seq. BMC Genom..

[102] Tayeh N., Aubert G., Pilet-Nayel M.-L., Lejeune-Hénaut I., Warkentin T.D., Burstin J. (2015). Genomic tools in pea breeding programs: Status and perspectives. Front. Plant Sci..

[103] Collard B.C.Y., Mackill D.J. (2008). Marker-assisted selection: An approach for precision plant breeding in the twenty-first century. Philos. Trans. R. Soc. B Biol. Sci..

[104] Desta Z.A., Ortiz R. (2014). Genomic selection: Genome-wide prediction in plant improvement. Trends Plant Sci..

[105] Tanksley S., Nelson J. (1996). Advanced backcross QTL analysis: A method for the simultaneous discovery and transfer of valuable QTL from unadapted germplasm into elite breeding lines. Theor. Appl. Genet..

[106] Alonso-Blanco C., Koornneef M., Stam P., Martinez-Zapater J., Salinas J. (1998). The use of recombinant inbred lines (RILs) for genetic mapping. Arabidopsis Protocols.

[107] Kooke R., Wijnker E., Keurentjes J.J. (2012). Backcross populations and near isogenic lines. Methods Mol. Biol..

[108] Yu J.M., Holland J.B., McMullen M.D., Buckler E.S. (2008). Genetic design and statistical power of nested association mapping in maize. Genetics.

[109] Bandillo N., Raghavan C., Muyco P.A., Sevilla M.A.L., Lobina I.T., Dilla-Ermita C.J., Tung C.-W., McCouch S., Thomson M., Mauleon R. (2013). Multi-parent advanced generation inter-cross (MAGIC) populations in rice: Progress and potential for genetics research and breeding. Rice.

[110] Acquaah G. (2012). Principles of Plant Genetics and Breeding.

[111] Hufford M.B., Xu X., van Heerwaarden J., Pyhajarvi T., Chia J.M., Cartwright R.A., Elshire R.J., Glaubitz J.C., Guill K.E., Kaeppler S.M. (2012). Comparative population genomics of maize domestication and improvement. Nat. Genet..

[112] Huang X.H., Kurata N., Wei X.H., Wang Z.X., Wang A., Zhao Q., Zhao Y., Liu K.Y., Lu H.Y., Li W.J. (2012). A map of rice genome variation reveals the origin of cultivated rice. Nature.

[113] Xu X., Liu X., Ge S., Jensen J.D., Hu F., Li X., Dong Y., Gutenkunst R.N., Fang L., Huang L. (2012). Resequencing 50 accessions of cultivated and wild rice yields markers for identifying agronomically important genes. Nat. Biotechnol..

[114] Hu Z.B., Zhang D., Zhang G.Z., Kan G.Z., Hong D.L., Yu D.Y. (2014). Association mapping of yield-related traits and SSR markers in wild soybean (*Glycine soja* Sieb. and Zucc.). Breed. Sci..

[115] Jaccoud D., Peng K.M., Feinstein D., Kilian A. (2001). Diversity arrays: A solid state technology for sequence information independent genotyping. Nucleic Acids Res..

[116] Miller M.R., Dunham J.P., Amores A., Cresko W.A., Johnson E.A. (2007). Rapid and cost-effective polymorphism identification and genotyping using restriction site associated DNA (RAD) markers. Genome Res..

[117] Pratap A., Kumar J., Pratap A., Kumar J. (2014). Alien gene transfer in crop plants: An introduction. Alien Gene Transfer in Crop Plants, Volume 1: Innovations, Methods and Risk Assessment.

[118] Xie W., Feng Q., Yu H., Huang X., Zhao Q., Xing Y., Yu S., Han B., Zhang Q. (2010). Parent-independent genotyping for constructing an ultrahigh-density linkage map based on population sequencing. Proc. Natl. Acad. Sci. USA.

[119] Pandey M.K., Roorkiwal M., Singh V.K., Ramalingam A., Kudapa H., Thudi M., Chitikineni A., Rathore A., Varshney R.K. (2016). Emerging genomic tools for legume breeding: Current status and future prospects. Front. Plant Sci..

[120] Huang X.H., Feng Q., Qian Q., Zhao Q., Wang L., Wang A.H., Guan J.P., Fan D.L., Weng Q.J., Huang T. (2009). High-throughput genotyping by whole-genome resequencing. Genome Res..

[121] Yu H.H., Xie W.B., Wang J., Xing Y.Z., Xu C.G., Li X.H., Xiao J.H., Zhang Q.F. (2011). Gains in QTL detection using an ultra-high density SNP map based on population sequencing relative to traditional RFLP/SSR markers. PLoS ONE.

[122] Iquira E., Humira S., François B. (2015). Association mapping of QTLs for sclerotinia stem rot resistance in a collection of soybean plant introductions using a genotyping by sequencing (GBS) approach. BMC Plant Biol..

[123] Manavalan L.P., Prince S.J., Musket T.A., Chaky J., Deshmukh R., Vuong T.D., Song L., Cregan P.B., Nelson J.C., Shannon J.G. (2015). Identification of novel QTL governing root architectural traits in an interspecific soybean population. PLoS ONE.

[124] Muñoz N., Qi X., Li M., Xie M., Gao Y., Cheung M., Wong F., Lam H. (2016). Improvement in nitrogen fixation capacity could be part of the domestication process in soybean. Heredity.

[125] Mkwaila W., Terpstra K.A., Ender M., Kelly J.D. (2011). Identification of QTL for agronomic traits and resistance to white mold in wild and landrace germplasm of common bean. Plant Breed..

[126] Mamidi S., Miklas P.N., Trapp J., Felicetti E., Grimwood J., Schmutz J., Lee R., McClean P.E. (2016). Sequence-based introgression mapping identifies candidate white mold tolerance genes in common bean. Plant Genome.

[127] Blair M.W., Iriarte G., Beebe S. (2006). QLT analysis of yield traits in an advanced backcross population derived from a cultivated Andean× wild common bean (*Phaseolus vulgaris* L.) cross. Theor. Appl. Genet..

[128] Blair M.W., Izquierdo P. (2012). Use of the advanced backcross-QTL method to transfer seed mineral accumulation nutrition traits from wild to Andean cultivated common beans. Theor. Appl. Genet..

[129] O’Rourke J.A., Iniguez L.P., Fu F., Bucciarelli B., Miller S.S., Jackson S.A., McClean P.E., Li J., Dai X., Zhao P.X. (2014). An RNA-seq based gene expression atlas of the common bean. BMC Genom..

[130] Crampton M., Sripathi V.R., Hossain K., Kalavacharla V. (2016). Analyses of methylomes derived from Meso-American common bean (*Phaseolus vulgaris* L.) using MeDIP-seq and whole genome sodium bisulfite-sequencing. Front. Plant Sci..

[131] Ariani A., y Teran J.C.B.M., Gepts P. (2016). Genome-wide identification of SNPs and copy number variation in common bean (*Phaseolus vulgaris* L.) using genotyping-by-sequencing (GBS). Mol. Breed..

[132] Perseguini J.M.K.C., Oblessuc P.R., Rosa J.R.B.F., Gomes K.A., Chiorato A.F., Carbonell S.A.M., Garcia A.A.F., Vianello R.P., Benchimol-Reis L.L. (2016). Genome-wide association studies of anthracnose and angular leaf spot resistance in common bean (*Phaseolus vulgaris* L.). PLoS ONE.

[133] Schröder S., Mamidi S., Lee R., McKain M.R., McClean P.E., Osorno J.M. (2016). Optimization of genotyping by sequencing (GBS) data in common bean (*Phaseolus vulgaris* L.). Mol. Breed..

[134] Wang D., Graef G., Procopiuk A., Diers B. (2004). Identification of putative QTL that underlie yield in interspecific soybean backcross populations. Theor. Appl. Genet..

[135] Winter S.M., Shelp B.J., Anderson T.R., Welacky T.W., Rajcan I. (2007). QTL associated with horizontal resistance to soybean cyst nematode in *Glycine soja* PI464925B. Theor. Appl. Genet..

[136] Zhang H., Li C., Davis E.L., Wang J., Griffin J.D., Kofsky J., Song B.-H. (2016). Genome-wide association study of resistance to soybean Cyst nematode (*Heterodera glycines*) HG type 2.5.7 in wild soybean (*Glycine soja*). Front. Plant Sci..

[137] Bajaj D., Das S., Upadhyaya H.D., Ranjan R., Badoni S., Kumar V., Tripathi S., Gowda C.L., Sharma S., Singh S. (2015). A genome-wide combinatorial strategy dissects complex genetic architecture of seed coat color in chickpec. Front. Plant Sci..

[138] Upadhyaya H.D., Bajaj D., Das S., Saxena M.S., Badoni S., Kumar V., Tripathi S., Gowda C., Sharma S., Tyagi A.K. (2015). A genome-scale integrated approach aids in genetic dissection of complex flowering time trait in chickpea. Plant Mol. Biol..

[139] Saxena M.S., Bajaj D., Das S., Kujur A., Kumar V., Singh M., Bansal K.C., Tyagi A.K., Parida S.K. (2014). An integrated genomic approach for rapid delineation of candidate genes regulating agro-morphological traits in chickpea. DNA Res..

[140] Srivastava R., Singh M., Bajaj D., Parida S.K. (2016). A high-resolution InDel (insertion–deletion) markers-anchored consensus genetic map identifies major QTLs governing pod number and seed yield in chickpea. Front. Plant Sci..

[141] Das S., Singh M., Srivastava R., Bajaj D., Saxena M.S., Rana J.C., Bansal K.C., Tyagi A.K., Parida S.K. (2016). MQTL-seq delineates functionally relevant candidate gene harbouring a major QTL regulating pod number in chickpea. DNA Res..

[142] Leal-Bertioli S.C., José A.C., Alves-Freitas D.M., Moretzsohn M.C., Guimarães P.M., Nielen S., Vidigal B.S., Pereira R.W., Pike J., Fávero A.P. (2009). Identification of candidate genome regions controlling disease resistance in *Arachis*. BMC Plant Biol..

[143] Leal-Bertioli S.C., Moretzsohn M.C., Roberts P.A., Ballén-Taborda C., Borba T.C., Valdisser P.A., Vianello R.P., Araújo A.C.G., Guimarães P.M., Bertioli D.J. (2016). Genetic mapping of resistance to *Meloidogyne arenaria* in *Arachis stenosperma*: A new source of nematode resistance for peanut. G3.

[144] Fonceka D., Tossim H.A., Rivallan R., Vignes H., Faye I., Ndoye O., Moretzsohn M.C., Bertioli D.J., Glaszmann J.C., Courtois B. (2012). Fostered and left behind alleles in peanut: Interspecific QTL mapping reveals footprints of domestication and useful natural variation for breeding. BMC Plant Biol..

[145] Burow M.D., Simpson C.E., Paterson A.H., Starr J.L. (1996). Identification of peanut (*Arachis hypogaea* L.) RAPD markers diagnostic of root-knot nematode (*Meloidogyne arenaria* (Neal) Chitwood) resistance. Mol. Breed..

[146] Fonceka D., Tossim H.-A., Rivallan R., Vignes H., Lacut E., de Bellis F., Faye I., Ndoye O., Leal-Bertioli S.C., Valls J.F. (2012). Construction of chromosome segment substitution lines in peanut (*Arachis hypogaea* L.) using a wild synthetic and QTL mapping for plant morphology. PLoS ONE.

[147] Fondevilla S., Satovic Z., Rubiales D., Moreno M.T., Torres A.M. (2008). Mapping of quantitative trait loci for resistance to *Mycosphaerella pinodes* in *Pisum sativum* subsp. *syriacum*. Mol. Breed..

[148] Weeden N.F. (2007). Genetic changes accompanying the domestication of *Pisum sativum*: Is there a common genetic basis to the “domestication syndrome” for legumes?. Ann. Bot..

[149] Saxena K., Ariyanayagam R., Reddy L. (1992). Genetics of a high-selfing trait in pigeonpea. Euphytica.

[150] Saxena K., Kumar R., Rao P. (2002). Pigeonpea nutrition and its improvement. J. Crop Prod..

[151] Saxena K., Kumar R., Latha K.M., Dalvi V. (2006). Commercial pigeonpea hybrids are just a few steps away. Indian J. Pulses Res..

[152] Saxena R.K., Cui X., Thakur V., Walter B., Close T.J., Varshney R.K. (2011). Single feature polymorphisms (SFPS) for drought tolerance in pigeonpea (*Cajanus* spp.). Funct. Integr. Genom..

[153] Tullu A., Bett K., Banniza S., Vail S., Vandenberg A. (2013). Widening the genetic base of cultivated lentil through hybridization of *Lens culinaris* “Eston” and *L. ervoides* accession IG 72815. Can. J. Plant Sci..

[154] Andargie M., Knudsen J.T., Pasquet R.S., Gowda B.S., Muluvi G.M., Timko M.P. (2014). Mapping of quantitative trait loci for floral scent compounds in cowpea (*Vigna unguiculata* L.). Plant Breed..

[155] Andargie M., Pasquet R.S., Gowda B.S., Muluvi G.M., Timko M.P. (2011). Construction of a SSR-based genetic map and identification of QTL for domestication traits using recombinant inbred lines from a cross between wild and cultivated cowpea (*V. unguiculata* (L.) Walp.). Mol. Breed..

[156] Andargie M., Pasquet R.S., Muluvi G.M., Timko M.P. (2013). Quantitative trait loci analysis of flowering time related traits identified in recombinant inbred lines of cowpea (*Vigna unguiculata*). Genome.

[157] Kongjaimun A., Kaga A., Tomooka N., Somta P., Shimizu T., Shu Y., Isemura T., Vaughan D.A., Srinives P. (2012). An SSR-based linkage map of yardlong bean (*Vigna unguiculata* (L.) Walp. subsp. *unguiculata* Sesquipedalis Group) and QTL analysis of pod length. Genome.

[158] Kongjaimun A., Kaga A., Tomooka N., Somta P., Vaughan D.A., Srinives P. (2012). The genetics of domestication of yardlong bean, *Vigna unguiculata* (L.) Walp. ssp. *unguiculata* cv.-gr. *sesquipedalis*. Ann. Bot..

[159] Kongjaimun A., Somta P., Tomooka N., Kaga A., Vaughan D.A., Srinives P. (2013). QTL mapping of pod tenderness and total soluble solid in yardlong bean [*Vigna unguiculata* (L.) Walp. subsp. *unguiculata* cv.-gr. *sesquipedalis*]. Euphytica.

[160] Varshney R.K. (2016). Exciting journey of 10 years from genomes to fields and markets: Some success stories of genomics-assisted breeding in chickpea, pigeonpea and groundnut. Plant Sci..

[161] Singh R., Sharma P., Varshney R.K., Sharma S., Singh N. (2008). Chickpea improvement: Role of wild species and genetic markers. Biotechnol. Genet. Eng. Rev..

[162] Srivastava R., Bajaj D., Malik A., Singh M., Parida S.K. (2016). Transcriptome landscape of perennial wild *Cicer microphyllum* uncovers functionally relevant molecular TAGs regulating agronomic traits in chickpea. Sci. Rep..

[163] Kujur A., Upadhyaya H.D., Bajaj D., Gowda C., Sharma S., Tyagi A.K., Parida S.K. (2016). Identification of candidate genes and natural allelic variants for QTLs governing plant height in chickpea. Sci. Res..

[164] Bajaj D., Srivastava R., Nath M., Tripathi S., Bharadwaj C., Upadhyaya H.D., Tyagi A.K., Parida S.K. (2016). Ecotilling-based association mapping efficiently delineates functionally relevant natural allelic variants of candidate genes governing agronomic traits in chickpea. Front. Plant Sci..

[165] Kudapa H., Azam S., Sharpe A.G., Taran B., Li R., Deonovic B., Cameron C., Farmer A.D., Cannon S.B., Varshney R.K. (2014). Comprehensive transcriptome assembly of chickpea (*Cicer arietinum* L.) using sanger and next generation sequencing platforms: Development and applications. PLoS ONE.

[166] Bertioli D.J., Seijo G., Freitas F.O., Valls J.F., Leal-Bertioli S.C., Moretzsohn M.C. (2011). An overview of peanut and its wild relatives. Plant Genet. Resour..

[167] Hong Y., Pandey M.K., Liu Y., Chen X., Liu H., Varshney R.K., Liang X., Huang S. (2015). Identification and evaluation of single-nucleotide polymorphisms in allotetraploid peanut (*Arachis hypogaea* L.) based on amplicon sequencing combined with high resolution melting (HRM) analysis. Front. Plant Sci..

[168] Li L., Redden R.J., Zong X., Berger J., Bennett S.J. (2013). Ecogeographic analysis of pea collection sites from China to determine potential sites with abiotic stresses. Genet. Resour. Crop Evol..

[169] Boutet G., Alves Carvalho S., Falque M., Peterlongo P., Lhuillier E., Bouchez O., Lavaud C., Pilet-Nayel M.-L., Rivière N., Baranger A. (2016). SNP discovery and genetic mapping using genotyping by sequencing of whole genome genomic DNA from a pea RIL population. BMC Genom..

[170] Sharpe A.G., Ramsay L., Sanderson L.-A., Fedoruk M.J., Clarke W.E., Li R., Kagale S., Vijayan P., Vandenberg A., Bett K.E. (2013). Ancient orphan crop joins modern era: Gene-based SNP discovery and mapping in lentil. BMC Genom..

[171] Saxena K. (2008). Genetic improvement of pigeonpea—A review. Trop. Plant Biol..

[172] Saxena R.K., Saxena K., Varshney R.K. (2010). Application of SSR markers for molecular characterization of hybrid parents and purity assessment of ICPH 2438 hybrid of pigeonpea [*Cajanus cajan* (L.) Millspaugh]. Mol. Breed..

[173] Bohra A., Jha R., Singh I.P., Pandey G., Pareek S., Basu P.S., Chaturvedi S.K., Singh N.P. (2017). Novel CMS lines in pigeonpea [*Cajanus cajan* (L.) Millspaugh] derived from cytoplasmic substitutions, and their effective restoration and deployment in hybrid breeding. Crop J..

[174] Saxena K., Sultana R., Mallikarjuna N., Saxena R., Kumar R., Sawargaonkar S., Varshney R. (2010). Male-sterility systems in pigeonpea and their role in enhancing yield. Plant Breed..

[175] Pazhamala L., Saxena R.K., Singh V.K., Sameerkumar C., Kumar V., Sinha P., Patel K., Obala J., Kaoneka S.R., Tongoona P. (2015). Genomics-assisted breeding for boosting crop improvement in pigeonpea (*Cajanus cajan*). Front. Plant Sci..

[176] Mallikarjuna N., Jadhav D., Reddy P. (2006). Introgression of *Cajanus platycarpus* genome into cultivated pigeonpea, *C. cajan*. Euphytica.

[177] Saxena K., Singh L., Reddy M., Singh U., Lateef S., Sharma S., Remanandan P. (1990). Intra species variation in *Atylosia scarabaeoides* (L.) Benth., a wild relative of pigeonpea (*Cajanus cajan* (L.) Millsp.). Euphytica.

[178] Boukar O., Fatokun C.A., Huynh B.-L., Roberts P.A., Close T.J. (2016). Genomic tools in cowpea breeding programs: Status and perspectives. Front. Plant Sci..

[179] Fatokun C.A., Singh B.B. (1987). Interspecific hybridization between *Vigna pubescens* and *V. unquiculata* (L.) Walp through embryo rescue. Plant Cell Tissue Organ Cult..

[180] Souleymane A., Aken’Ova M., Fatokun C., Alabi O. (2013). Screening for resistance to cowpea aphid (*Aphis craccivora* koch) in wild and cultivated cowpea (*Vigna unguiculata* L. Walp.) accessions. Int. J. Sci. Environ. Technol..

[181] HarvEST. http://harvest.ucr.edu/.

[182] Wyrwa K., Książkiewicz M., Szczepaniak A., Susek K., Podkowiński J., Naganowska B. (2016). Integration of *Lupinus angustifolius* L. (narrow-leafed lupin) genome maps and comparative mapping within legumes. Chromosome Res..

[183] Parra-González L.B., Aravena-Abarzúa G.A., Navarro-Navarro C.S., Udall J., Maughan J., Peterson L.M., Salvo-Garrido H.E., Maureira-Butler I.J. (2012). Yellow lupin (*Lupinus luteus* L.) transcriptome sequencing: Molecular marker development and comparative studies. BMC Genom..

[184] Gao L.-L., Hane J.K., Kamphuis L.G., Foley R., Shi B.-J., Atkins C.A., Singh K.B. (2011). Development of genomic resources for the narrow-leafed lupin (*Lupinus angustifolius*): Construction of a bacterial artificial chromosome (BAC) library and BAC-end sequencing. BMC Genom..

[185] Tian L., Peel G.J., Lei Z., Aziz N., Dai X., He J., Watson B., Zhao P.X., Sumner L.W., Dixon R.A. (2009). Transcript and proteomic analysis of developing white lupin (*Lupinus albus* L.) roots. BMC Plant Biol..

[186] Lulsdorf M.M., Ferrie A., Slater S.M.H., Yuan H.Y., Pratap A., Kumar J. (2014). Methods and Role of Embryo Rescue Technique in Alien Gene Transfer. Alien Gene Transfer in Crop Plants, Volume 1: Innovations, Methods and Risk Assessment.

[187] Hajjar R., Hodgkin T. (2007). The use of wild relatives in crop improvement: A survey of developments over the last 20 years. Euphytica.

[188] Warschefsky E., Penmetsa R.V., Cook D.R., von Wettberg E.J. (2014). Back to the wilds: Tapping evolutionary adaptations for resilient crops through systematic hybridization with crop wild relatives. Am. J. Bot..

[189] Hawkes J. (1977). The importance of wild germplasm in plant breeding. Euphytica.

[190] Zhang H., Mittal N., Leamy L.J., Barazani O., Song B.H. (2017). Back into the wild-apply untapped genetic diversity of wild relatives for crop improvement. Evol. Appl..

[191] Upadhyaya H.D., Dwivedi S.L., Ambrose M., Ellis N., Berger J., Smýkal P., Debouck D., Duc G., Dumet D., Flavell A. (2011). Legume genetic resources: Management, diversity assessment, and utilization in crop improvement. Euphytica.

[192] Kilian B., Graner A. (2012). NGS technologies for analyzing germplasm diversity in genebanks. Brief. Funct. Genom..

[193] Van Treuren R., van Hintum T.J. (2014). Next-generation genebanking: Plant genetic resources management and utilization in the sequencing era. Plant Genet. Resour..

[194] Ma C., Zhang H.H., Wang X. (2014). Machine learning for big data analytics in plants. Trends Plant Sci..

[195] Maxted N., Dulloo E., V Ford-Lloyd B., Iriondo J.M., Jarvis A. (2008). Gap analysis: A tool for complementary genetic conservation assessment. Divers. Distrib..

[196] Ramírez-Villegas J., Khoury C., Jarvis A., Debouck D.G., Guarino L. (2010). A gap analysis methodology for collecting crop genepools: A case study with *Phaseolus* beans. PLoS ONE.

[197] Dempewolf H., Eastwood R.J., Guarino L., Khoury C.K., Müller J.V., Toll J. (2014). Adapting agriculture to climate change: A global initiative to collect, conserve, and use crop wild relatives. Agroecol. Sust. Food.

[198] Castañeda-Álvarez N.P., Khoury C.K., Achicanoy H.A., Bernau V., Dempewolf H., Eastwood R.J., Guarino L., Harker R.H., Jarvis A., Maxted N. (2016). Global conservation priorities for crop wild relatives. Nat. Plants.

